# Isolated hypoxic pelvic perfusion combined with electroporation is a valid alternative to traditional therapies for anal squamous cell carcinoma: a case study

**DOI:** 10.3389/fonc.2025.1644317

**Published:** 2025-08-27

**Authors:** Karl R. Aigner, Kornelia Aigner, Marc J. H. Hendrikx, Abderrahmane Bekrentchir, Hansjörg Aust

**Affiliations:** ^1^ Department of Surgical Oncology, Medias Klinikum Burghausen, Burghausen, Germany; ^2^ Department of Tumor Biology, Medias Klinikum Burghausen, Burghausen, Germany; ^3^ Department of Anesthesiology, Medias Klinikum Burghausen, Burghausen, Germany

**Keywords:** anal cancer, isolated perfusion, intra-arterial chemotherapy, electrochemotherapy, squamous cell carcinoma, toxic side effects, quality of life

## Abstract

**Introduction:**

We report a series of four patients with HPV-positive squamous cell carcinoma of the anus: two with non-previously treated grade G3 malignancies, and two with relapsing G2 tumors in progression after chemoradiation.

**Method:**

Chemo-naive patients were treated with isolated hypoxic pelvic perfusion, whereas the two patients with local recurrences both received isolated hypoxic pelvic perfusion and concomitant reversible electroporation.

**Results:**

The two patients without prior therapy showed a complete clinical and pathological response, one after two and one after three therapies. In the two patients with local recurrences, a clinico-pathological complete remission occurred after two isolated perfusions with electroporation. The two techniques combined produce highly effective drug exposure that leads to complete remission; subsequent blood purification by chemofiltration avoids toxic side effects and cumulative toxicity. The therapies caused no side effects affecting quality of life. No recurrences occurred in the observation period of 48, 27, 10 and 8 months.

**Conclusion:**

Isolated hypoxic pelvic perfusion with electroporation is an effective, low-toxicity valid alternative to traditional therapies for the treatment of anal squamous cell carcinoma.

## 1 Introduction

Squamous cell carcinoma of the anus is a very rare but extremely debilitating tumor and only accounts for about 2% of all gastrointestinal tumors (1–4). However, the incidence appears to have increased slightly over the last decade. Due to the high chemosensitivity of squamous cell carcinoma chemoradiation is the treatment of choice compared to invasive surgical procedures (5, 6).

Nevertheless, the optimal treatment remains controversial given the sometimes exorbitant toxicity of chemotherapeutical drugs such as mitomycin and radiation therapy.

Many attempts have been made to reduce toxicity, with intensity-modulated radiation therapy bringing some improvement, whereas a quantitative reduction in chemotherapy by lowering the effective levels also reduces the therapeutic effect.

Hypoxic pelvic perfusion increases the sensitivity of some chemotherapeutic agents (e.g. cis platinum). Additionally limiting the chemotherapy exposure to the pelvic region reduces systemic toxicity which in turn minimizes systemic side effects.

Early case reports suggest that hypoxic pelvic perfusion can lead to high local response rates of rectal cancer (7).

However, hypoxic pelvic perfusion alone is limited by the challenge of ensuring complete tumor coverage.

Electroporation increases cell membrane permeability therefore enhancing chemotherapy drug effectiveness. Several clinical studies have shown that electroporation can enhance the local response however it is often limited by the depth of the tumor and the ability to treat larger more deeply seated tumors combining hypoxic pelvic perfusion and electroporation overcomes the limitation of each approach (8).

Astoundingly higher cytostatic drug levels and therefore greater effectiveness can be achieved through isolated pelvic regional perfusion with comparatively lower cytostatic drug dosages.

We present a series of four HPV-positive patients with anal cancer, two with malignancy grade G3 primary tumors, and two with recurrent G2 tumors. In the latter two cases there was a relapse after primary radiochemotherapy.

## 2 Materials and methods

### 2.1 Isolated hypoxic pelvic perfusion technique with chemofiltration

Under general anesthesia, two pneumatic cuffs are placed around the proximal thighs. Below the inguinal ligament, the femoral artery and vein are exposed through a longitudinal incision and cannulated with two triple-lumen stop-flow balloon catheters under systemic heparinization.

**Figure 1:**
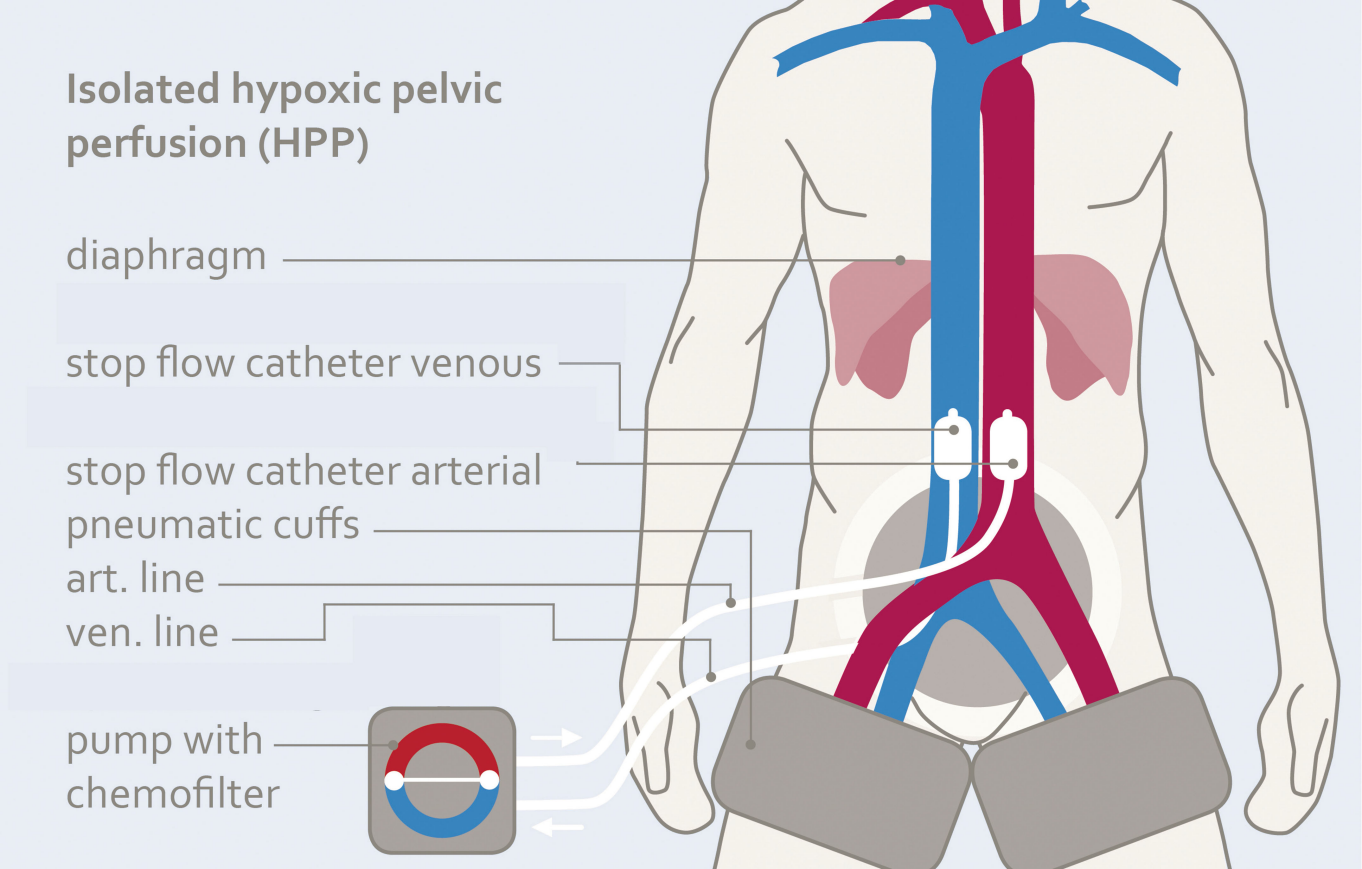
Scheme of isolated hypoxic pelvic perfusion (HPP) for short-term intraaortic infusion of cytotoxic agents Mitomycin, Doxorubicin and Cisplatin.

**Figure 2:**
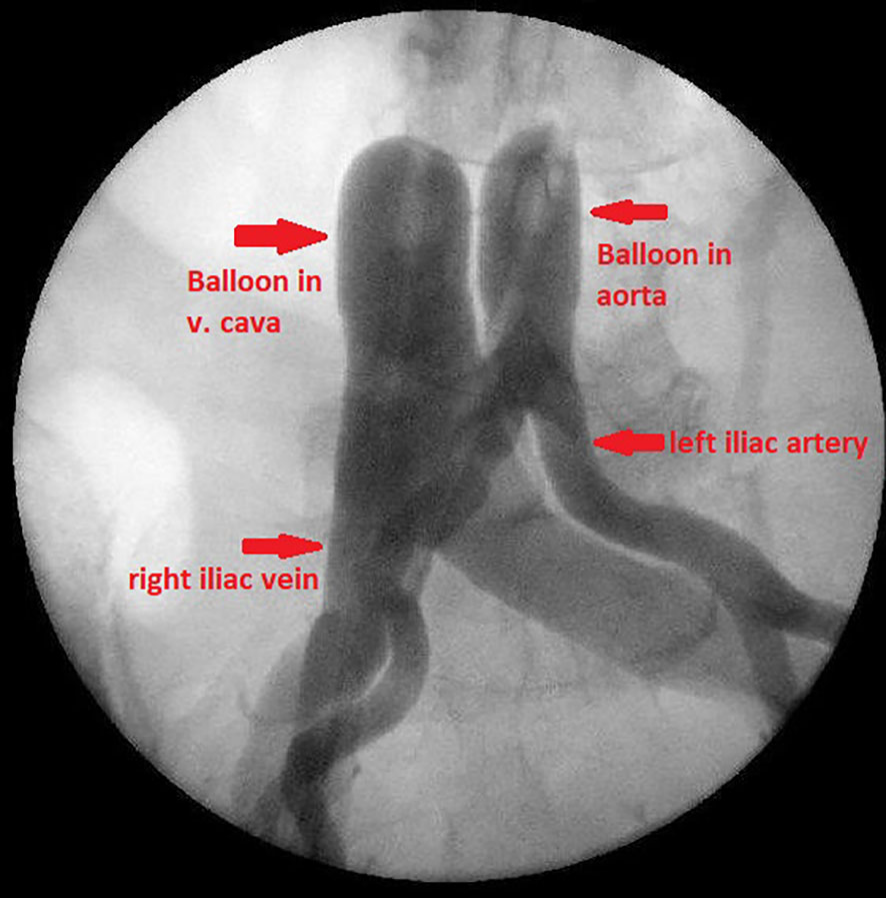
Balloon blocking of aorta and vena cava proximal to the bifurcations.

The balloons are placed proximal to the bifurcation of the aorta and vena cava under contrast medium control (**Figure 1**). The correct position of the balloons in the aorta and vena cava is ensured by brief blocking and photo documentation (**Figure 2**). Both balloons are then immediately released and the patient is ventilated with 100% oxygen for approximately three minutes to increase oxygen saturation before starting therapy.

**Figure 3:**
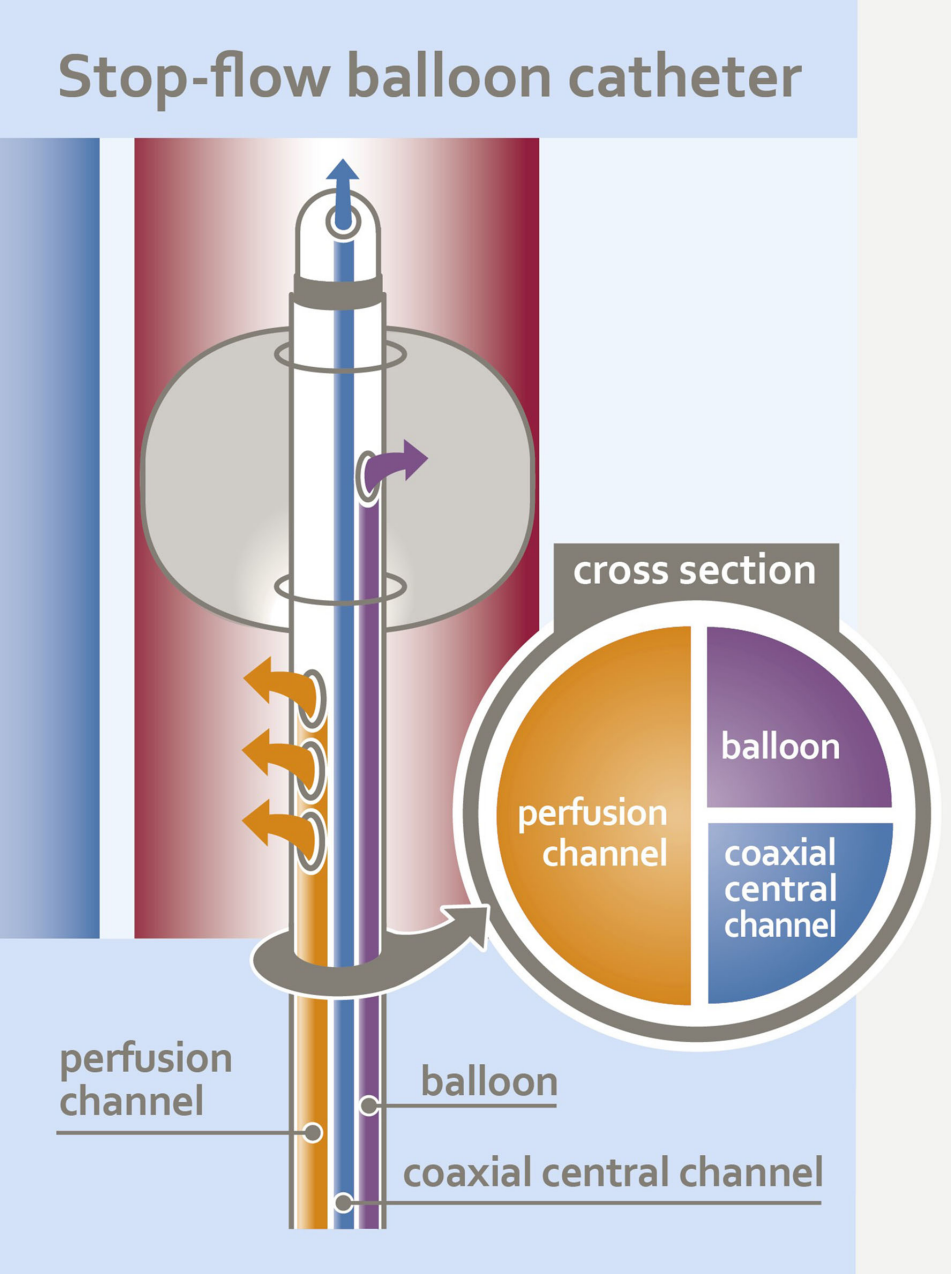
Schematic design of the triple-lumen stop-flow ballon catheter.

**Figure 4:**
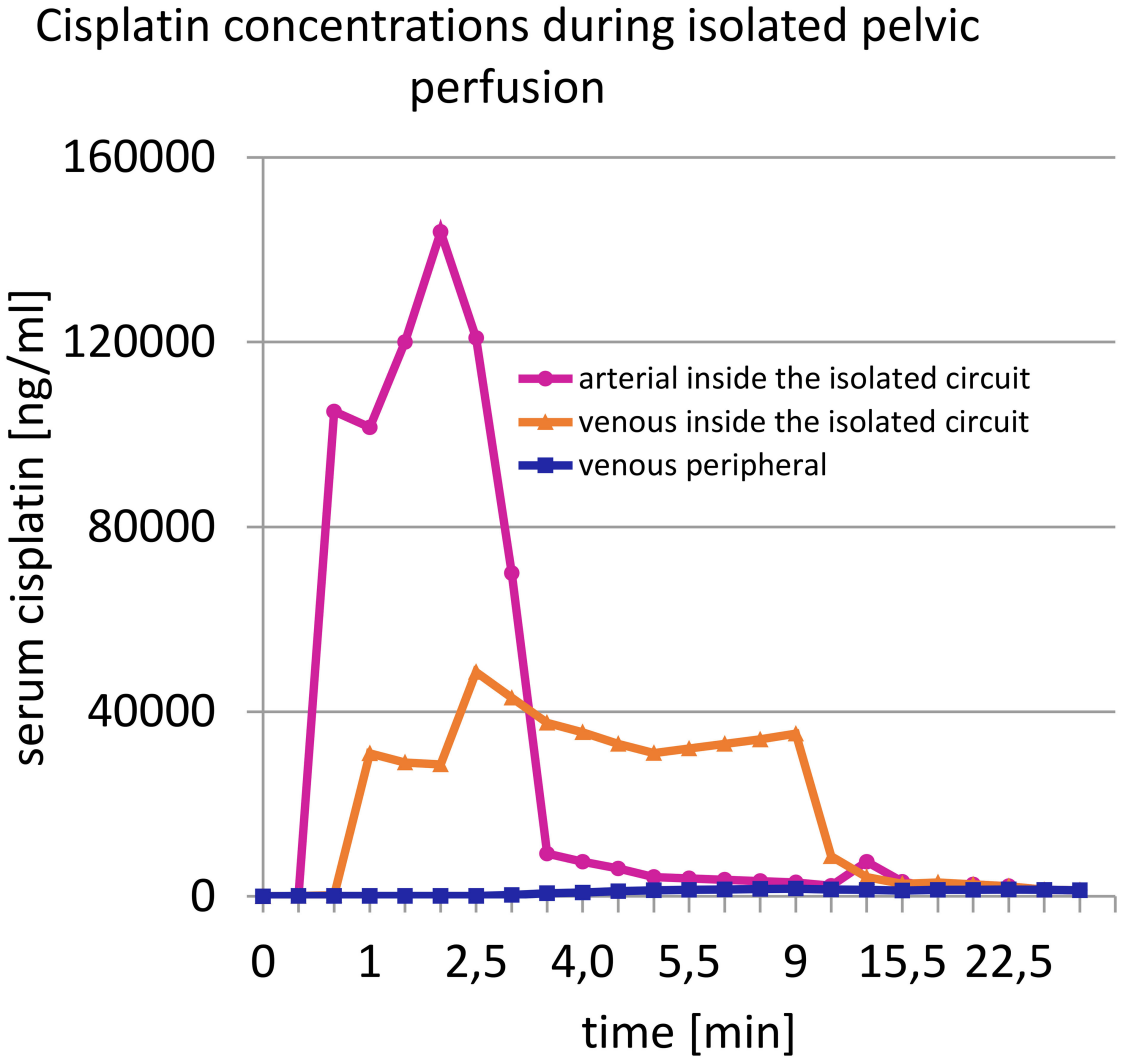
Concentration curve of cisplatin in the arterial and venous line at start of the stop-flow phase of isolated perfusion.

Chemotherapy is administered through rhythmic, pulsatile injections via the large perfusion channel of the aortic balloon catheter (**Figure 3**) over one to one and a half minutes to achieve high first-pass drug concentrations (**Figure 4**). Immediately afterwards, both balloons are blocked with diluted contrast medium. A five-minute so-called stop-flow phase creates a high first- pass effect and therefore a high uptake of cytostatics in the tumor area due to the high initial oxygen saturation. Isolated hypoxic perfusion is maintained for 15 minutes. The stop-flow phase is included in the total time of isolated hypoxic perfusion. Both balloons are then unblocked and the blood in the open system is filtered through capillary filters via the catheters still in place for approximately another 30 to 40 minutes until a filtrate volume of minimum 4 liters is reached. Finally, the two balloon catheters are removed, the vessels repaired with running sutures and the wound is closed.

### 2.2 Electrochemotherapy

**Figure 5:**
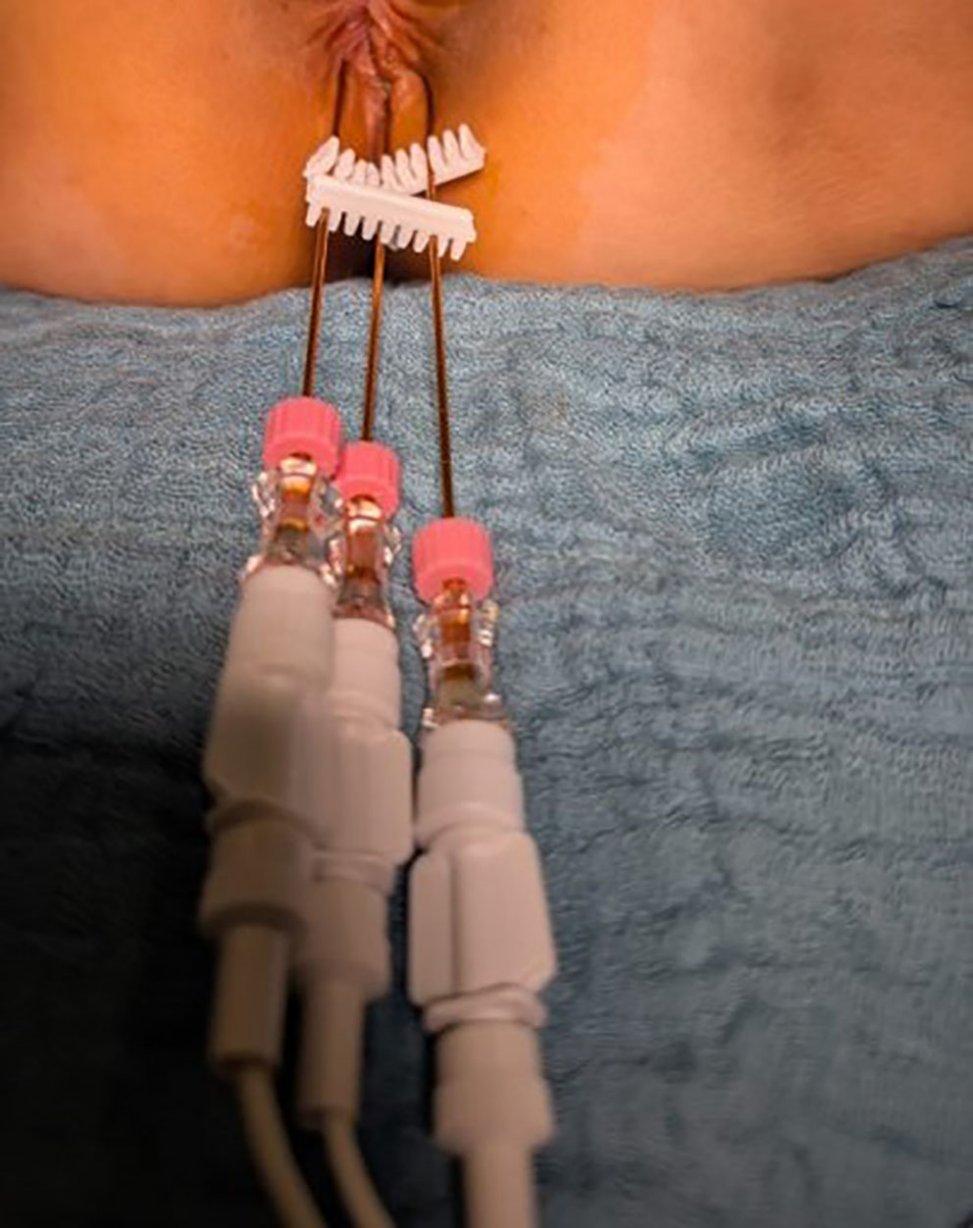
Positioning of the electrodes in anal carcinoma between 5 and 7 o’ clock in lithotomy position.

When combining isolated perfusion with reversible electroporation, three to four VGD- electrodes (needle diameter 1,2 mm, length 20 cm, active part 3 cm) (IGEA) are first placed under direct vision in the lithotomy position, close to the tumor extent (**Figure 5**), and connected to the Cliniporator VITAE (IGEA) pulse generator for adaptive electroporation. The patient is then placed in a normal supine position, the groin area is disinfected, and the femoral arteries and veins are cannulated (see 2.1). The chemotherapy drugs are administered during the electrical pulses.

## 3 Results

Patient characteristics according to diagnosis, histology, previous treatment and type of therapy are listed in **Table 1**.

**Table 1:** Patient characteristics.

	Pretreatment	Diagnosis	Histology	Therapy	Follow-up
Case 1 50 yo female	None	22x16 mm bleeding tumor 5-9o’clock	G3 basaloid SCC	3x HPP 50 CCP 30 DOX 10 MMC +filtration	48 M
Case 2 59 yo female	RT 5 fractions	Recurrence 6-9o’clock Bleeding tumor	Non keratinizing SCC T2N0M0	HPP x2 50CCP 30 DOX 10 MMC + filtration Iliac art infusion x3: 1x CCP + mitoxantrone 2x CCP + DOX HPP +electrochem 50 CCP 30 Dox 10 MMC	10M
Case 3 48 yo female	None	Ulcerated narrowing + infiltration post. vag. wall	G3 SCC 58x40x54 mm	2x HPP 50 CCP 30 DOX 10 MMC	27 M
Case 4 62 yo Female	-chemoRT -R1 local resection	Extensive local recurrence	G2 SCC 40x30 mm	HPP – filtration Electrochemotherapy 70 CCP 30 DOX 10 MMC	8 M

CCP, cis-platinum; DOX, doxorubicin; HPP, hypoxic pelvic perfusion; M, Months; MMC, mitomycine C; RT, radiotherapy; SCC, squamous cell carcinoma.

### 3.1 Case 1

The first case concerns a non-pretreated 50-year-old female patient with a 22 to 16 mm, occasionally bleeding squamous cell tumor near the skin margin, extending from 5 to 9 o’clock in the lithotomy position. Histologically, it was a malignancy grade G3 basaloid squamous cell carcinoma. Imaging revealed no evidence of lymph node metastases. The patient underwent a total of three cycles of isolated hypoxic pelvic perfusion, each containing 50 mg of cisplatin, 30 mg of doxorubicin, 10 mg of mitomycin, followed by chemofiltration. No systemic or local adverse effects were observed. Two days after the first treatment, the tumor had already become significantly softer and flatter. At the time of the second treatment, three weeks later, the patient experienced neither pain nor bleeding.

Before the third treatment, the tumor was no longer clearly demarcated on MRI, and two weeks later, on July 20, 2021, local excision of the former tumor region revealed a histologically complete remission. The patient has currently been disease-free in complete remission for 48 months.

### 3.2 Case 2

The second case concerns a 59-year-old female patient who was initially diagnosed with a T2N0M0 non-keratinizing bleeding anal squamous cell carcinoma (KI 67 of 50%) in November 2022 and received five fractions of radiotherapy. In January 2023 she presented with a local recurrence which was located at the 6–9 o’clock position and extended up to 4 cm into the anal canal. She underwent her first isolated hypoxic pelvic perfusion with chemofiltration (HPP-F) receiving 50 mg cisplatin, 30 mg doxorubicin, and 10 mg mitomycin. Before the second treatment, three weeks later in February 2023, with the same technique and dosage, the tumor size had decreased by more than half both macroscopically and on palpation. Six days after the second HPP-F, only two residual small indurations were visible, and the final treatment was scheduled three weeks later. Due to unforeseen personal circumstances, the patient was unable to attend the appointment. A follow-up examination two months later revealed increasing local induration, suggesting a recurrence. Three consecutive angiographic infusions into the iliac artery with one cisplatin/mitoxantrone and two cisplatin/doxorubicin infusions failed to result in significant improvement. Only when the patient consented to isolated pelvic perfusion with reversible electroporation using the cytostatic combination of 50 mg of cisplatin, 30 mg of doxorubicin, and 10 mg of mitomycin in September 2024 did rapid tumor reduction occur. The patient did not suffer any significant toxicities due to subsequent chemofiltration with capillary filters. Re-excision of the previously necrotic tumor area demonstrated a histologically complete remission. The patient is currently free of symptoms or relapses since the last therapy 10 months ago.

### 3.3 Case 3

The third case concerns a non-pretreated 48-year-old female patient with an ulcerated grade G3 squamous cell carcinoma of the anus (5.8 × 4 × 5.4 cm) showing narrowing growth and infiltration of the posterior vaginal wall.

She experienced severe pain when sitting and walking. One day after the first isolated perfusion with 50 mg cisplatin, 25 mg doxorubicin, and 10 mg mitomycin and chemofiltration in March 2023, the patient was pain-free without evidence of toxicity.

Prior to the second isolated perfusion using the same dosage, no tumor was palpable locally; only 90% regressive tumor residue was found on biopsy at the former infiltration site of the posterior vaginal wall. The patient did not attend a third appointment for treatment. A follow- up examination one year later revealed a complete remission by biopsy.

The patient is currently disease-free in complete remission for 27 months.

### 3.4 Case 4

This 62-year-old female patient was diagnosed with grade G2 squamous cell carcinoma measuring 4x3 cm in January 2024.

In February/March 2024, chemoradiotherapy was performed without apparent clinical response, followed by a local R1 resection. When extensive local recurrence occurred, the patient was advised to undergo rectal amputation, which she declined. She underwent hypoxic isolated pelvic perfusion (HPP-F) to avoid rectal amputation.

Due to the advanced stage of the disease with a rapid progressive recurrence, we combined HPP-F and reversible electroporation with 70 mg of cisplatin, 30 mg of doxorubicin and 10 mg of mitomycin in October 2024. After the treatment, pain was significantly reduced, and the patient’s general condition improved considerably.

After the second HPP-F in November 2024 without any toxic side-effects, repeated biopsies revealed complete histological remission, currently for 8 months.

## 4 Discussion

Historically, the primary treatment for anal cancer was abdominoperineal resection (APR), a radical surgery leaving patients with a permanent colostomy. This was standard through the mid-20^th^ century, though it had significant drawbacks: high morbidity, poor quality of life, and a risk of cancer recurrence. The 5-year survival rate was approximately 50% (3, 9). When used as a salvage surgery after failed CRT the 2-year OS is ~60%, but 5-year OS drops to ~24.5%.

A major breakthrough came in the late 1970s and early 1980s with the introduction of the Nigro protocol, a pioneering approach combining radiation therapy with chemotherapy (5-fluorouracil and mitomycin C) (10–12). This approach demonstrated that many patients could achieve complete remission without surgery. As a result, chemoradiation became the standard of care, allowing for organ preservation. Local control is achieved in 68 - 84%, colostomy-free survival in 65 - 75% and 5-year OS in 65 – 79% (4, 13–17).

Acute toxicities are common: hematologic (cytopenias), mucocutaneous, gastrointestinal, genitourinary symptoms. Late effects may include chronic bowel dysfunction, sexual dysfunction, infertility, or chronic pain. Even local excision alone of stage I anal cancer can be effective and curative. However, careful follow-up is required because the 5-year progression-free survival after combination with radiochemotherapy is slightly better at 91 versus 83% for local excision (18). Radiochemotherapy regimens consisting of 5-fluorouracil and mitomycin and concomitant radiation achieved stage-dependent 5-year survival rates of up to 90% and sphincter preservation of up to 80% (12, 19) while avoiding rectal amputation (20, 21). Despite these excellent clinical results (15), the severe toxicity with mandatory interruption of therapy clouds the overall picture in approximately half of the cases.

Precisely because of the good clinical results in the face of intolerable side-effects such as nephrotoxicity or pulmonary fibrosis, attempts were made to modify the treatment regimens, using immunotherapy (22–24), new drugs such as capecitabine or paclitaxel, or intensity – modulated radiotherapy (25–27), to improve quality of life while maintaining clinical outcomes.

Isolated perfusion therapy can be viewed as a further step in terms of both intensifying therapy and minimizing or eliminating side effects. It can be applied to all body regions (28) or segments in the form of an isolated circuit.

For hypoxic perfusion, which we prefer to use, mitomycin is best suited as a very potent chemotherapeutic agent for gastrointestinal tumors because of its up to tenfold increase in cytotoxicity under hypoxic conditions. The same applies to doxorubicin. The efficacy of Cisplatin is not affected. All other common chemotherapy drugs have reduced cytotoxicity under hypoxia (29).

The high effective concentrations of cytostatics are achieved on the one hand by the low blood volumes in the perfusion circuit, but above all by the application of the cytostatics as a short-term arterial infusion into the isolated perfusion circuit. The high influx concentration of chemotherapy drugs over seven to twelve minutes leads to high uptake in the tumor tissue. This short-term intra-arterial infusion procedure has been shown to be effective in squamous cell carcinomas such as head and neck cancer (30, 31), in advanced cervical cancer (32), and more recently in anal cancer. Even in advanced osteosarcoma, complete remission can be achieved due to high local drug exposure using a technical variant (33).

In the two previously untreated patients, three and two isolated perfusion therapies were necessary; in the case of previous radiochemotherapy and recurrences as in cases two and four, repeated intra-arterial infusion alone was not sufficient because the local cytostatic exposure was too low. Only isolated pelvic perfusion in combination with electroporation achieved a histological complete remission (**Table 2**) due to increased cytostatic drug uptake via temporarily porous tumor cell membranes as a result of the electrical impulses (34).

**Table 2:** Results according to therapeutic options.

Treatment option	Colostomy free rate	Functional outcome	Toxicity/morbidity	5-year survival
APRA (primary)	0%	Poor permanent stoma	High surgical morbidity	~50%
APRA (salvage)	0%	Poor permanent stoma	Very high morbidity (~80%)	24 – 60%
CRT (5-FU+MMC)	65 – 80%	Preserved sphincter most	Acute: hematolog/GI/dermatolog some late	65 – 90%
Locoreg CT+/-RT	100% (this series)	Preserved sphincter	None	TBD

The dosages of chemotherapeutic agents used by us under locoregional perfusion chemotherapy are very different from those under systemic chemotherapy. The smaller treated volume and, above all, the only short-term intra-arterial application of the chemotherapeutic agent generate the required local exposure with the necessary high concentration. Therefore, the effectiveness of the therapy cannot be assessed based on the total dose used but rather based on the effective concentration over time. The total dose of cisplatin used in the present isolated pelvic perfusions is only 50 to 70 mg, of mitomycin 10, max 15 mg with, however, very effective local exposure with short-term intra-arterial infusion into the perfusion circuit.

On the one hand, chemofiltration after application of the cytostatics reduces the low toxicity of low-dose chemotherapeutic agents, but primarily prevents cumulative toxicity from occurring too quickly, especially from substances with cumulative toxicity such as mitomycin and doxorubicin.

Even though the number of cases is of course far too small and no binding statement can be made, it turns out that all four patients tolerated the therapies largely without any symptoms and no relevant adverse events in the sense of systemic toxicity occurred. The recurrence- free times in full remission are currently 48, 27, 10 and 8 months.

The present case series was simply intended to demonstrate that it is fundamentally possible to apply a proven, highly effective approach such as mitomycin compounds to squamous cell carcinoma, in this case anal carcinoma, using regional therapeutic methods that are modified to largely avoid disruptive and distressing adverse events. Isolated hypoxic pelvic perfusion and electrochemotherapy for anal cancer with short-term arterial infusion of chemotherapy drugs into the arterial perfusion line together produce highly effective drug exposure leading to complete remission. The subsequent blood purification using chemofiltration largely avoids toxic side effects and cumulative toxicity.

## 5 Conclusion

Isolated hypoxic pelvic perfusion combined with electroporation is an effective, low-toxicity valid alternative to traditional therapies for the treatment of anal squamous cell carcinoma.

## Data Availability

The data analyzed in this study is subject to the following licenses/restrictions: Protected patient data. Requests to access these datasets should be directed to info@prof-aigner.de.
